# Evaluation of expansion and crossing profile characteristics of micro laser powder bed fusion-manufactured coronary stents in a rat model

**DOI:** 10.1038/s41598-025-27767-3

**Published:** 2025-11-18

**Authors:** Pakhwan Nilcham, Xuezhi Cao, Nicole Schaaps, Lukas Masseling, Carolina Bienzeisler, Rahma Shahin, Fabian Kießling, Marek Weiler, Liguo Zhao, Felix Jan Vogt, Anne Turoni-Glitz

**Affiliations:** 1 Department of Cardiology, Angiology, and Internal Intensive Medicine, Uniklinik RWTH Aachen, Pauwelsstraße 30, 52074 Aachen, Germany; 2 Wolfson School of Mechanical, Electrical and Manufacturing Engineering, Loughborough University, Epinal Way, Loughborough, LE11 3TU UK; 3 Fraunhofer-Institute for Laser Technology ILT, 52074 Aachen, Germany; 4 Aixway3D GmbH, 52074 Aachen, Germany; 5 Institute for Experimental Molecular Imaging, Uniklinik RWTH Aachen University, 52074 Aachen, Germany; 6 School of Energy and Power Engineering, Nanjing University of Aeronautics and Astronautics, Nanjing, 210016 China

**Keywords:** Coronary artery disease, Coronary stents, µ-LPBF stents, Additive manufacturing, Personalized medicine, In vivo evaluation, Cardiology, Engineering, Materials science, Medical research

## Abstract

Micro-laser powder bed fusion (µ-LPBF) technology holds significant potential for fabricating coronary stents tailored to an individual’s anatomy, potentially addressing the issue of in-stent restenosis (ISR). Despite several studies on µ-LPBF stents, no experimental work has investigated their implantability in a rat model. This pilot study fabricated two types of µ-LPBF stents from 316 L stainless steel (316 L SS) and cobalt-chromium (CoCr) and assessed their implantability in the rat abdominal aorta through *post-mortem* experiments and one in vivo implantation of a 316 L SS stent. Expansion behavior was evaluated in vitro through free expansion tests and within healthy and 70% stenosed artificial arteries. We demonstrated successful implantation of µ-LPBF stents in the rat aorta. Both stent types showed sufficient deliverability and could be advanced through the femoral artery to the abdominal aorta without fracturing. The stents maintained their integrity under external tissue compression, as evidenced by histological and µCT images. Greater expansion was observed in 316 L SS stents compared to CoCr stents in both in vitro and in vivo experiments. Non-uniform expansion was observed for both µ-LPBF stent types, with *post-mortem* and artificial arteries experiments showing an opposite pattern to the ‘dog bone’ phenomenon. These results demonstrate the feasibility of µ-LPBF stent fabrication and successful implantation in rats, highlighting the potential of µ-LPBF as a promising approach for producing personalized stents or stents with fully customized geometries.

## Introduction

Coronary artery disease (CAD) remains one of the major causes of global morbidity and mortality^1^. Percutaneous coronary intervention represents the current standard therapeutic procedure for CAD. Ever since the first bare metal stents (BMS) were introduced more than three decades ago, drug-eluting stents (DES) and bioresorbable stents (BRS) have been developed, and stent materials, designs, and production techniques have been evolving over time to overcome unfavorable clinical complications such as in-stent restenosis (ISR) and stent thrombosis (ST)^2^. Various types of stents made from different materials, such as metal-based or polymer-based stents have been evaluated in broad research studies. While polymeric stents have superior flexibility compared to metallic stents, they require thicker stent struts to maintain radial strength equal to metallic stents^3^. However, strut thickness has been demonstrated to be associated with ISR^4^. Therefore, at present, commercially available stents are only metallic-based and have been predominantly used in clinical practice. Due to their production via laser cutting of microtubes, commercial stents are generally limited to standard, non-complex designs^5^.

Currently, all commercially available stents feature uniform designs in both the longitudinal and circumferential directions, disregarding the substantial variability in lesion profiles among individual patients. As addressed in our previous research^6^, stents with lesion-specific designs can offer several advantages over conventional uniform designs. By matching the unique geometry and curvature of each patient’s vessel, customized stents can improve mechanical compatibility, ensure optimal expansion and apposition, and minimize stress concentration. As demonstrated previously^6^, tailored stents were associated with increased luminal gain, improved luminal shape, and reduced arterial damage, which is directly linked to the risk of ISR^7^.

Among patients, however, there are individual differences in coronary artery anatomy and in lesion morphology, with individual geometrical and physical characteristics of the plaque and patchy calcification patterns. Lesion complexity including vessel tortuosity and calcification has been demonstrated to be a risk factor for ISR and ST^8,9^. Stent size also plays a significant role in ISR. While undersizing of stents can lead to stent malapposition or stent under-expansion, oversizing of stents causes vessel wall injury contributing to ISR^10^.

Patient-specific stent designs tailored to the individual lesion geometry and texture might be a way to overcome these issues. However, conventional laser cutting technologies are not suited to fabricate patient- or lesion-specific stents with complex and non-uniform geometries, due to technical constraints and high production costs^5^.

Micro Laser Powder Bed Fusion (µ-LPBF) technology, an additive manufacturing (AM) technique, has gained significant attention in personalized stent research due to its ability to surpass the limitations of conventional laser-cutting techniques, as well as earlier generations of AM technologies such as Selective Laser Melting (SLM) and Laser Powder Bed Fusion (LPBF). The µ-LPBF process operates in a similar fashion to a conventional LPBF. Using these techniques, metal powders are selectively melted with a high-energy focal laser beam to create the desired structure^11^. However, µ-LPBF incorporates more advanced process parameters, including a smaller laser beam size (~ 25 μm), finer powder particle size (less than 10 μm), and a thinner layer thickness (~ 10 μm)^12^. These advancements enable the production of a micro-complex structure with a feature size as small as 30 μm, while conventional LPBF usually offers a size around 200 μm with a surface roughness of 7–20 μm^11^. Therefore, the µ-LPBF technique is particularly well-suited for fabricating vascular stents, especially due to its ability to produce thinner stent struts with diameter ≤ 100 μm.

Several studies have employed AM techniques for manufacturing vascular stents and explored stent designs, material characterization, and in vitro mechanical properties. For example, in 2017, Demir et al.^13^ proposed a prototype stent design for the AM technique and demonstrated its feasibility in cobalt-chromium (CoCr) stent production. Additionally, the group demonstrated the feasibility of using conventional electrochemical polishing to improve the surface quality of AM-manufactured stents. In 2019, the design rules of AM stents were proposed by Finazzi et al.^14^ which were subsequently implemented in the production of CoCr stents, resulting in favorable expansion and flexibility. Various types of metallic powders, such as 316 L stainless steel (316 L SS) and nickel-titanium alloy (NiTi), have been utilized for fabricating AM stents, with a focus on assessing manufacturing feasibility and mechanical properties^15–17^.

Beyond mechanical performance, a stent must also demonstrate excellent implantability and deliverability. This means the stent must be sufficiently flexible to pass through the complex, tortuous anatomy of the vessel and be able to conform to the shape of the vessel upon expansion. However, to the best of our knowledge, no experimental studies investigating the implantability of µ-LPBF stents, either *post-mortem* or in vivo, have been reported.

In this pilot study, two types of metallic powders, 316 L SS and CoCr, were used to fabricate stents using the µ-LPBF technique. These substances are commonly used for commercial stents due to their high radial strength, corrosion resistance, and biocompatibility^18^. We sought to evaluate the implantability of µ-LPBF stents in a rat model, both *post-mortem* and in vivo in a living rat. Micro-computed tomography (µCT) and histological analysis were conducted, followed by morphometric evaluation. Furthermore, we assessed the expansion behavior of µ-LPBF stents in vitro in free expansion experiments and by using artificial arteries with 0% and 70% cross-sectional stenosis.

## Methods

### µ-LPBF stent manufacturing

Stents were designed using an advanced computer-aided design software (Siemens NX 11). In line with the recommendations for AM of stents^13^, a closed-cell design was chosen. The stent design was composed of crowns and flex connectors commonly found on other stent designs.

**Fig. 1:**
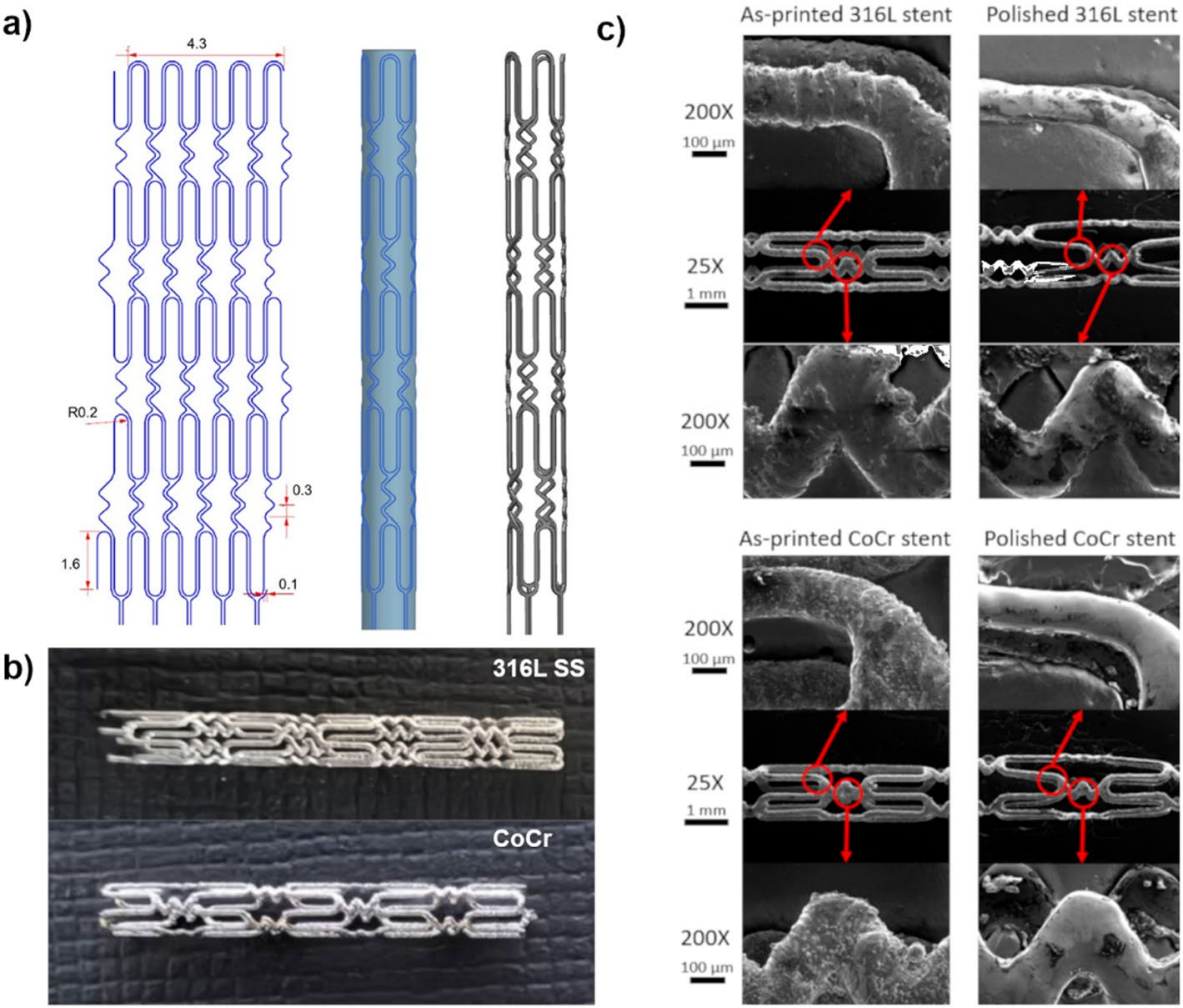
Design and manufacturing of µ-LPBF stents. (a) Steps of creating the 3D stent model: generating a 2D sketch (left), wrapping the 2D sketch around a tube (middle), cutting the tube according to the design and adding a thickness to obtain the final 3D stent model (right). (b) µ-LPBF fabricated U-bend stent with zig-zag connectors. (c) Scanning electron microscopy images of as-printed and polished 316 L SS and CoCr stents at 25 X and 200 X magnifications.

First, a computer-aided design model of the stent was created. The two-dimensional stent sketch was characterized by key design features of commercially available stents including Cypher (Cordis Corporation), Xience (Abbott Vascular) and Endeavor/Resolute (Medtronic) (Fig. 1a, left). Using the wrap-up tool in Siemens NX11, the 2D stent sketch was then wrapped on a tubular element to achieve the final tube shape of the stent (Fig. 1a, middle). The cells were produced in closed profiles by cutting the tubular element based on the design and leaving only the stent geometry. Finally, a thickness was added to the stent geometry to obtain a 3D stent model (Fig. 1a, right) for easy removal of the stent from the substrate. Stents were produced with support legs which were cut off after the µ-LPBF process (Fig. 1b). The stent was designed with a diameter of 1.36 mm, and a length of 10.5 mm. Strut thickness was 100 μm, close to the strut thickness of commercial stents, facilitating crimping and subsequent expansion process.

The µ-LPBF fabrication was conducted using an Aixway Precision 100 machine equipped with a 200 W Yb-fiber laser power source (Fraunhofer ILT, Germany). The machine is capable of focusing the laser beam to approximately 25 μm, and is therefore able to produce micro-structures with a size as small as 30 μm. The process used very fine powders with a particle diameter of less than 10 μm for both 316 L stainless steel and cobalt-chromium powders, combined with a thin layer thickness of 10 μm and modulated laser radiation. The fabrication was carried out under high purity argon atmosphere with an oxygen level below 50 ppm. An unheated stainless-steel plate was used as substrate. In the Aixway 3D standard µ-LPBF exposure process, a hatch-contour exposure was adopted, with a hatch spacing of 40 μm, as described previously^19^. The line energy inputs (combinations of laser power and scan speed) for the contour and hatch were set at 0.124 J/mm and 0.035 J/mm, respectively.

The overall diameter of the as-fabricated stent (*n* = 9) was measured as 1.289 ± 0.125 mm and 1.352 ± 0.027 mm (mean ± SD; Fig. 1c) for 316 L SS and CoCr stents, respectively, with a strut thickness of 140 μm; showing a discrepancy for the designed geometry. The surface roughness of the stent was measured with a non-contact focus variation microscopy (Alicona Infinite), with arithmetical average height (Sa) value of 4.16 μm and 5.02 μm and arithmetical average roughness (Ra) values of 5.757 μm and 6.571 μm, for 316 L SS and CoCr stents, respectively. Electrochemical polishing (ECP) was then carried out to further improve the stent surface as well as to reduce the stent size to match the original design.

### Free expansion test

A total of six stents (316 L SS *n* = 3, CoCr *n* = 3) were used. The stents were positioned onto the balloon catheter in a manner that the distal part of the stent (i.e. the side where the supporting legs were removed) faced the distal part of the balloon. The stents were crimped onto a commercial balloon catheter (TREK OTW Coronary Dilatation Catheter, 2.5 mm x 12 mm) with a diameter of 0.7 mm using a hand crimper (HH100 SS, Machine Solution Inc., USA ). The stents were placed under a light microscope (Leica DMI-3000, Wetzlar) and expanded at room temperature by inflating the balloon catheter in 1 atm increments using a manual inflator (Medex MEDFLATOR II Inflation Device). After each increment, the balloon catheter was deflated, and images were taken at the proximal, middle, and distal parts of the stent. Pressure was increased from 2 to 12 atm. The diameter of the stent was measured at each section of the stent (a minimum of seven measurements per stent in total) using Diskus software (Version 4.80, C. Hilgers Technisches Büro, Königswinter, Germany). The mean diameter of stent and standard error of the mean (SEM) at each stent part were calculated using GraphPad Prism (version 10). The data are represented as mean ± SEM.

### Expansion test in artificial artery model

A total of 12 artificial arteries were manufactured from 805 addition-cure silicone by Cixi Jiawang Metal Products Co. Ltd. All silicone arteries were fabricated with an outer diameter of 3.6 mm and a length of 30 mm. Six of the 12 silicone arteries had a wall thickness of 0.8 mm and an inner diameter of 2.0 mm, representing a healthy state, while the remaining six silicone arteries had a wall thickness of 1.5 mm and an inner diameter of 0.6 mm, corresponding to a diseased state with 70% cross-sectional stenosis. Wall thicknesses of healthy mock arteries were comparable to those of human coronary arteries, based on reported measurements of intima and media (0.3 mm ± 0.1 mm), and the adventitial layer (0.5 mm ± 0.2 mm)^20^. The stenosis rate was calculated by following formula (Eq. 1):

1 $$S=100\% *\frac{{ID - I{D_1}}}{{ID}},$$

in which *S*, $ID$ and $I{D_1}$ representing stenosis rate, inner diameter of healthy artery and inner diameter of diseased artery, respectively.

**Fig. 2:**
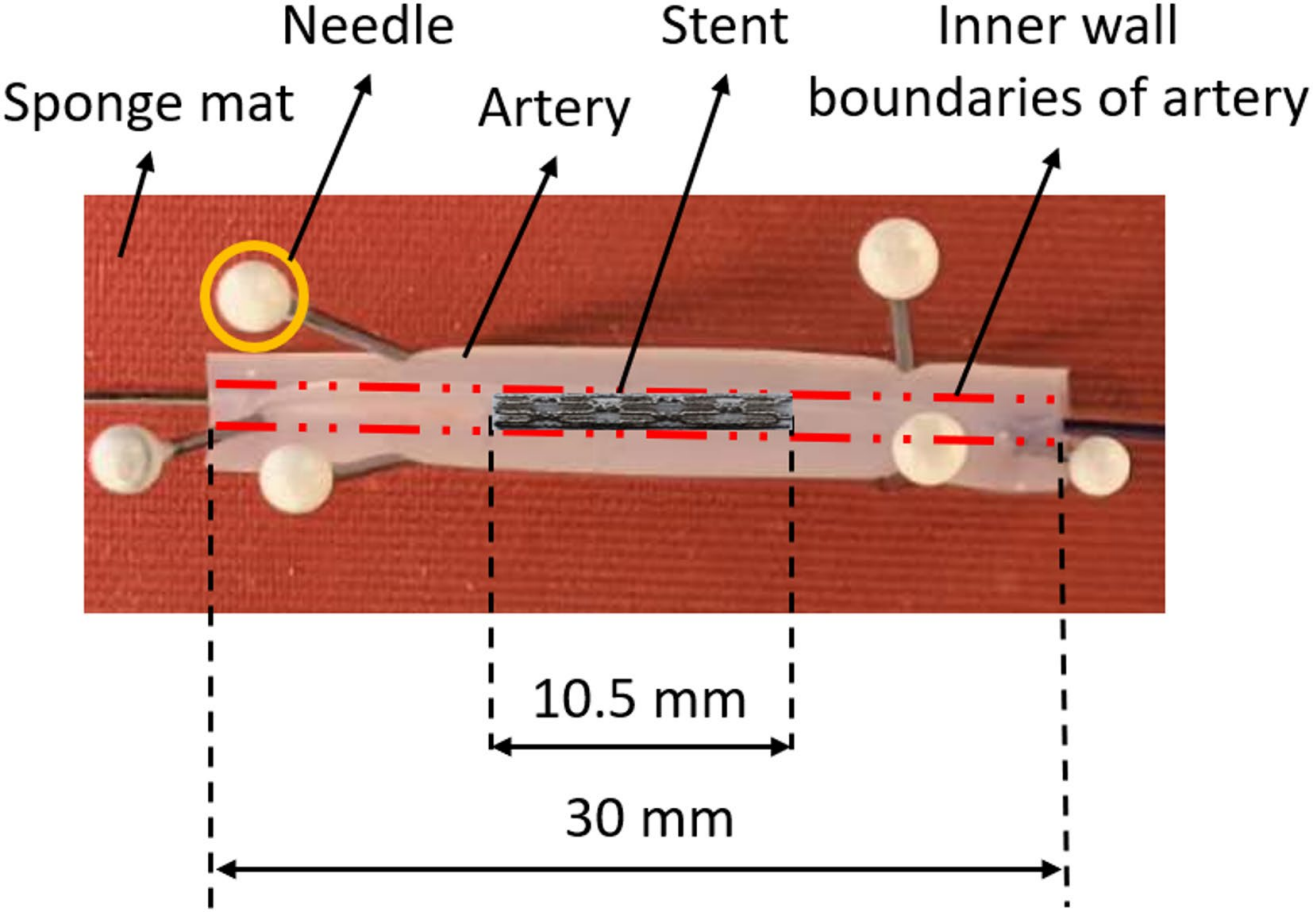
Stent positioned in a silicone artificial artery.

Stents were crimped onto a commercial balloon catheter (TREK OTW Coronary Dilatation Catheter, 2.5 mm x 12 mm) using a hand crimper (HH100 SS, Machine Solution Inc., USA). After crimping, the stent was inserted into the silicone artery, which was placed on a sponge mat and fixed by needles (Fig. 2). Expansion tests were performed with six 316 L SS and with six CoCr stents, with three of each stent type being implanted into healthy arteries and three of each stent type being implanted into stenosed silicone arteries.

The stent was expanded using a balloon with incremental pressure from 2 atm to 12 atm, in 1 atm steps, under an optical microscope (Smartscope Flash 200). The outer diameter of the stented artery was measured at each pressure level based on the optical images taken during the expansion process. Measurements were taken at the proximal, middle and distal parts of the stent. Direct measurement of the stent outer diameter was not feasible because the silicone artificial arteries were not completely transparent, and the stent contours appeared blurred in the images. Therefore, the inner and outer diameters of the mock arteries were used as indirect parameters to evaluate stent expansion behaviour, providing a practical surrogate for assessing stent expandability and uniformity under controlled experimental conditions. Each expansion experiment was repeated three times. At each pressure increment, the expanded artery was held for 10s, and two measurements were taken at each location using the optical microscope. The average diameter and standard deviation (SD) at each pressure level were calculated for the proximal, middle and distal parts. Finally, the data were recorded and analysed as line chart with standard deviations.

### Stent implantation in a rat model, micro-computed tomography, and histomorphometric evaluation

#### Stent implantation

A rat model was used to assess the implantability and expansion behavior of the µ-LPBF stents. First, stent performance was evaluated in a *post-mortem* setting, after the animal was euthanized (A4) in accordance with § 4 para. 3 of the Animal Protection Act of 18 May 2006 in the currently valid version. A total of four rats were used for µ-LPBF stent implantation, with two stents of each type (316 L SS, CoCr).

**Fig. 3:**
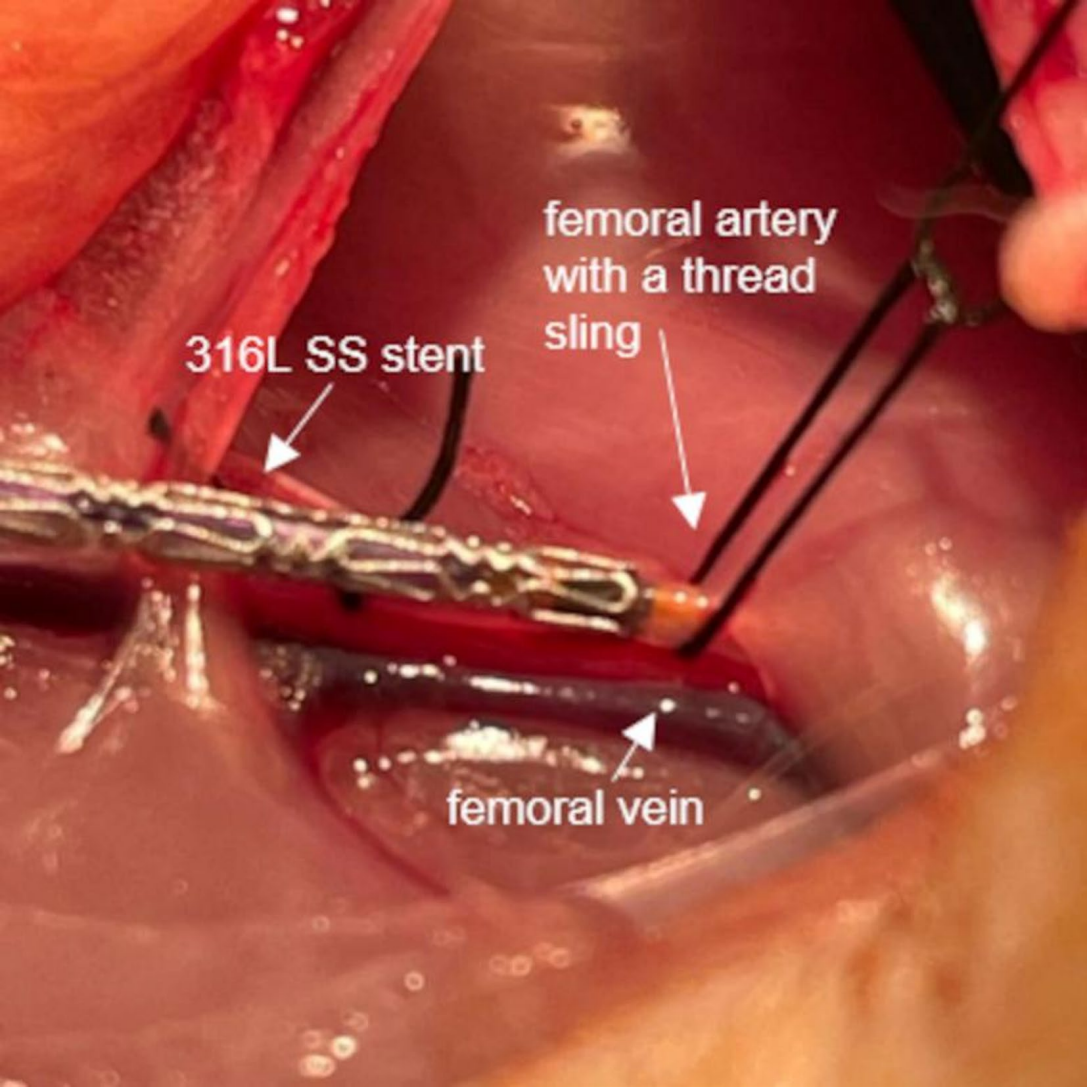
Insertion of a 316 L SS stent through a femoral artery incision.

Subsequently, one 316 L SS stent implanted in a living rat with approval from the Ministry of Nature, Environment and Country Development (Recklinghausen, Germany; AZ 81-02.04.2018.A23801). The rat was euthanized one day post-implantation using an overdose of isoflurane in accordance with approved protocols. All procedures were carried out in accordance with the ARRIVE guidelines and relevant guidelines and regulations.

Stent implantation in rats was performed as described previously^21^. Prior to in vivo implantation, the µ-LPBF stents were cleaned with 70% ethanol. Using a hand crimper (HH100 SS, Machine Solution Inc., USA), stents were crimped onto commercial balloons (TREK RX Coronary Dilation Catheter, 2.5 mm x 15 mm or 12 mm for *post-mortem* implantation, and 2.25 mm for in vivo implantation), with the distal end of the stent aligned with the distal end of the balloon. The stent was inserted using a transfemoral access and deployed in the abdominal aorta, below the renal arteries and above the aortic bifurcation (Fig. 3). Different inflation pressures were applied according to stent type. Three 316 L SS stents (two *post-mortem*; one in vivo) were implanted at 8 atm (*post-mortem*) and 10 atm (in vivo), whereas CoCr stents (both *post-mortem*) required higher pressures; one CoCr stent was implanted at 10 atm and one CoCr stent at 11 atm.

Following implantation, the aorta was explanted either immediately (*post-mortem* experiments) or one day after stent implantation (in vivo experiments) and fixed in 4% formaldehyde solution for further processing.

#### Micro-computed tomography

µCT was performed using a Bruker Skyscan 1272 (SkyScan, Belgium) scanner. The outer diameter of the deployed stents was assessed in cross-sectional views using Imalytics preclinical software (Gremse-IT)^22^, and measurements were performed in perpendicular lines from one abluminal surface of the stent to the opposite abluminal surface. The diameter of each stent was measured at the proximal, middle, and distal part, and two measurements per cross-section were taken. A total of 11 cross-sections per stent were analyzed.

#### Histomorphometric evaluation

Following µCT scanning, the stented aorta was processed for histomorphometrical evaluation. The aorta was embedded in methylmethacrylate (Technovit 9100, Morphisto, Offenbach am Main) and cut into sections of 30–50 micrometer thickness using a sawing-and-grinding technique^23^. Sections were polished and stained with Giemsa staining. Microscopical images were taken using a light microscope equipped with a digital camera (2.5x magnification, Leica DMI-3000, Wetzlar). Assessment of the outer stent diameter was performed by an independent observer using DISKUS image analysis software (version 4.80, C. Hilgers Technical Bureau, Königswinter). Two measurements from one abluminal stent strut surface to the opposite abluminal surface were made per section, at least 4 sections per stent.

## Results

### Surface and dimensional refinement via electrochemical Polishing

ECP significantly improved the surface quality of the µ-LPBF stents, with a marked reduction in discrepancies for overall diameter and strut size, achieving values within 5% of the original design. After ECP, the mean surface roughness (Ra) across multiple stents was reduced from 6.57 ± 1.51 μm to 2.924 ± 1.59 μm for CoCr stents, and from 5.253 ± 0.59 μm to 2.162 ± 0.85 μm for 316 L SS stents. This corresponds to a decrease of 67% for CoCr stents and 49% for 316 L SS stents compared to the as-printed values. In addition, strut thickness was reduced from 158.97 ± 4.78 μm to 123.69 ± 3.44 μm for CoCr stents, and from 144.38 ± 3.63 μm to 100.53 ± 4.43 μm for 316 L SS stents. The final lengths of the stents were measured as 10.82 ± 0.03 mm for CoCr and 10.88 ± 0.05 mm for 316 L SS (mean ± SEM).

### Free expansion test

**Fig. 4:**
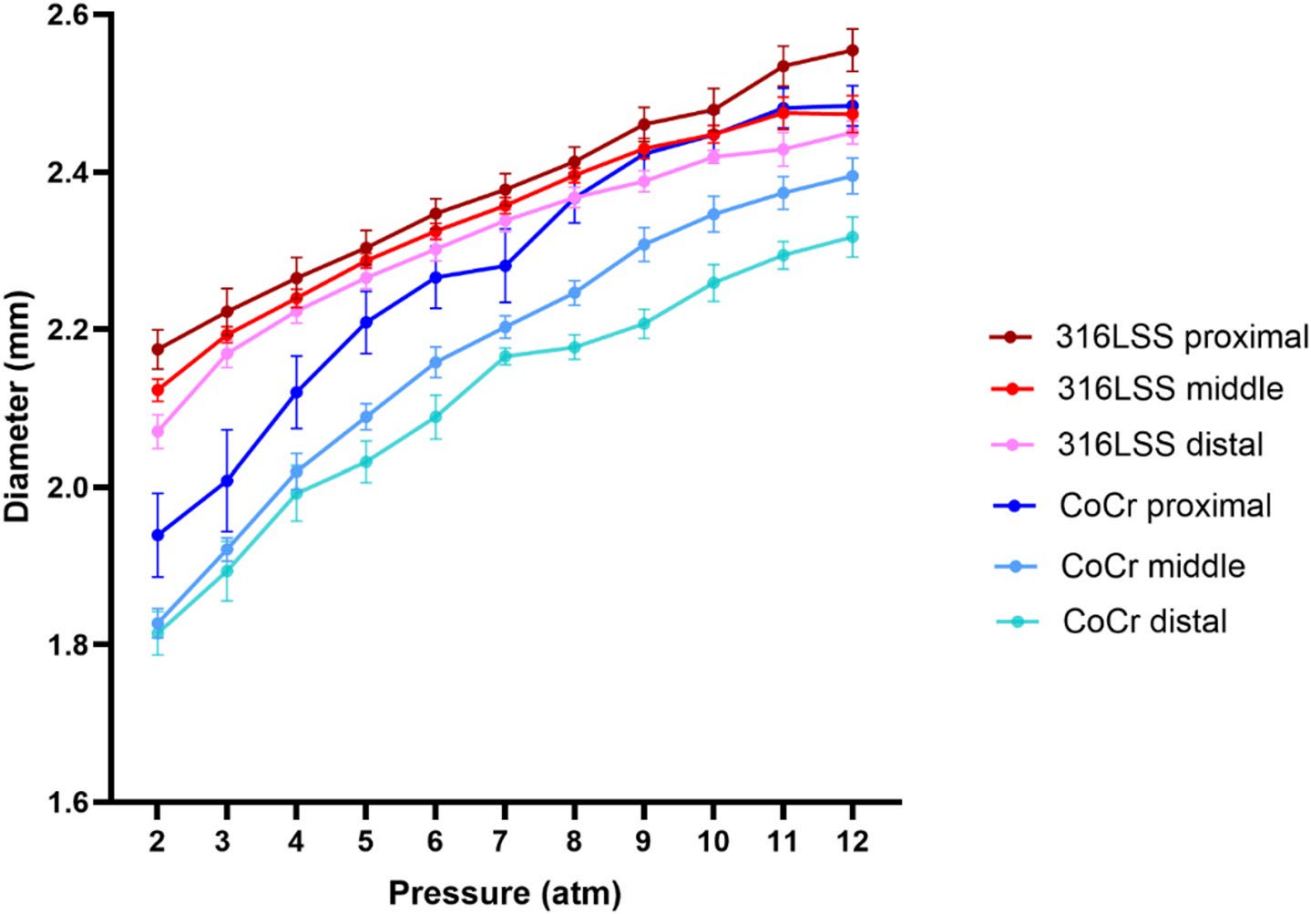
Mean diameters (± SEM) of 316 L SS and CoCr µ-LPBF stents at the proximal, middle, and distal parts during balloon expansion in 1 atm increments from 2 atm to 12 atm.

To assess free-expansion behavior in vitro, µ-LPBF stents were expanded from 2 to 12 atm. The mean outer diameters at the proximal, middle, and distal part of µ-LPBF stents, are presented in Fig. 4. The results are presented as mean ± SEM. With similar inflation pressure, 316 L SS stents had a greater increase in diameter compared to the CoCr stents. Both 316 L SS and CoCr stents demonstrated a deformation in the form of non-uniform expansion along their length, with the proximal part exhibiting the largest expansion, followed by the middle and the distal parts. This deformation was more prominent in CoCr stents compared to 316 L SS stents, especially at the proximal part, which demonstrated significant deviation from the middle and distal parts.

Under-expansion was observed in both 316 L SS and CoCr stents, as they failed to reach the desired diameter (2.5 mm) at the nominal pressure (8 atm) of the balloon. In particular, 316 L SS stents expanded from 2.12 ± 0.02 mm at 2 atm to 2.39 ± 0.01 mm at 8 atm, while the CoCr stents expanded from 1.87 ± 0.04 mm at 2 atm to a smaller diameter of 2.25 ± 0.02 mm at 8 atm. By increasing the pressure to 12 atm, the mean diameter of the 316 L SS stent increased to 2.49 ± 0.01 mm, and to 2.39 ± 0.02 mm for the CoCr stent. As a result, both 316 L SS and CoCr µ-LPBF stents did not reach the desired diameter at either nominal or higher pressure, albeit with superior expansion performance of the 316 L SS stents.

### Expansion tests in artificial arteries

**Fig. 5:**
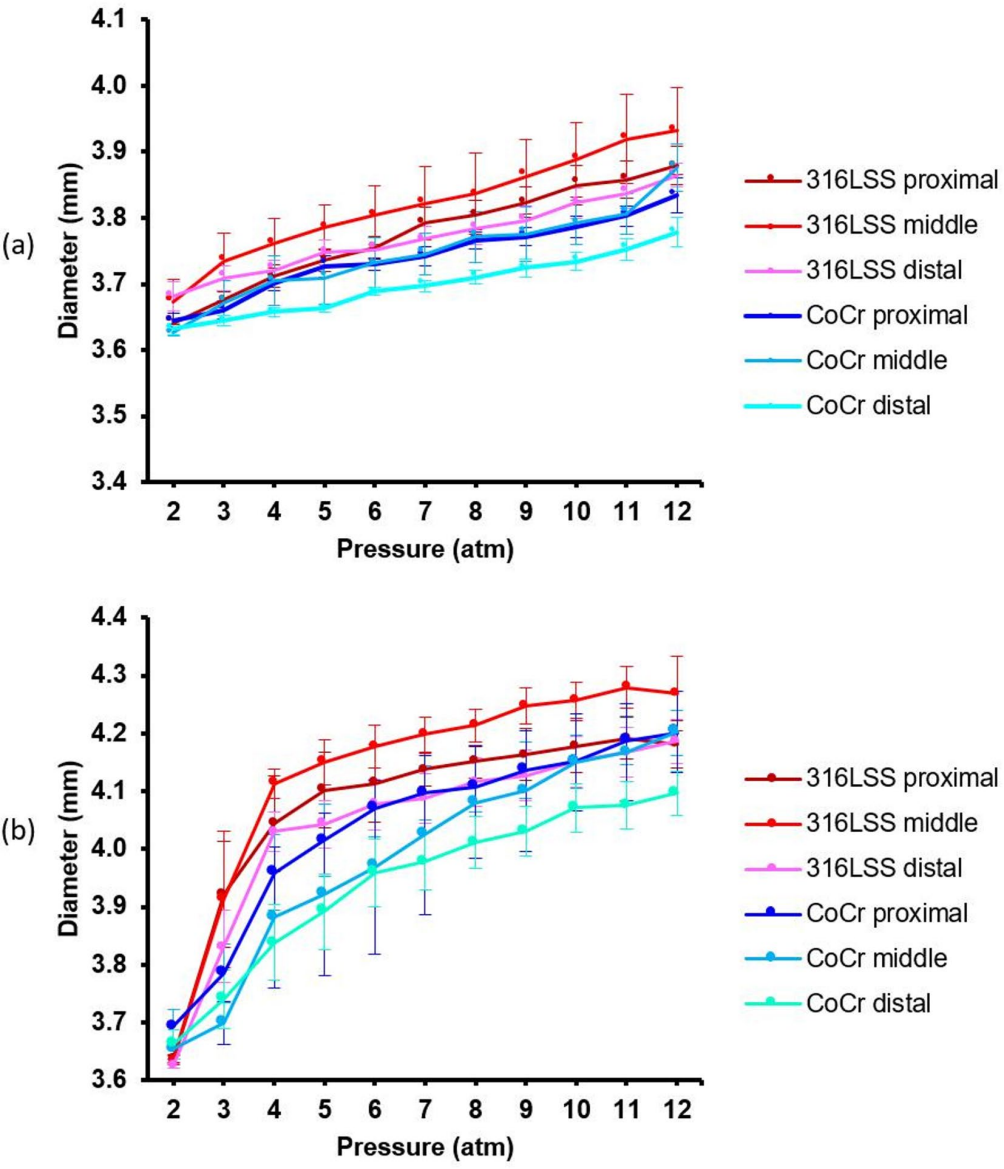
Expansion behavior of µ-LPBF stents in artificial arteries. The outer diameter of the artificial arteries is expressed as mean ± SD. (a) Stents expanded in healthy arteries. (b) Stents expanded in diseased arteries with 70% luminal stenosis.

Next, we evaluated stent expansion in artificial silicone arteries representing “healthy” and 70% stenosed “diseased” states. Direct measurement of the stent outer diameter was not feasible due to the partial opacity of the silicone arteries and blurred stent contours. Therefore, inner and outer diameters of the arteries were used as surrogate metrics. Measurements at proximal, middle, and distal parts were used to evaluate expansion uniformity along the stent length with increasing pressure from 2 atm to 12 atm (Fig. 5).

Stent expansion was generally greater in 316 L SS stents than CoCr stents, at identical inflation pressures (Fig. 5a and b). In healthy arteries (Fig. 5a), the outer diameters of CoCr and 316 L SS stented arteries were comparable at each increment, whereas in diseased arteries, 316 L SS stents showed more pronounced expansion (Fig. 5b). Additionally, the outer diameters of stented diseased arteries were larger than those of stented healthy arteries, which may be due to the non-uniform mechanical properties of stents associated with the AM process.

For 316 L SS stents, the greatest expansion occurred at the middle part, followed by the proximal and distal parts. In healthy arteries, the outer diameter increased from 3.67 mm ± 0.04 mm at 2 atm to 3.89 mm ± 0.07 mm at 12 atm (approximately 0.15 mm less than free expansion), while the inner diameter increased from 2 mm to 2.22 mm, (approximately 11% increase). In diseased arteries with 70% stenosis, the outer diameter increased from 3.63 mm ± 0.01 mm to 4.08 mm ± 0.01 mm as pressure increased from 2 atm to 4 atm, and further gradually from 4.08 ± 0.01 mm to 4.2 ± 0.1 mm from 4 atm to 12 atm. The inner diameter expanded from 0.6 mm to 1.17 mm (approximately 95% increase). Percentage increases in outer diameter at 12 atm were 8.17% for the healthy artery and 16.89% for the diseased artery, respectively.

Expansion behavior of the CoCr stent in artificial arteries was comparable to its free expansion behavior, with the proximal part exhibiting the largest expansion, followed by the middle and distal parts. Measurement deviations for the CoCr stent were similar to those for 316 L SS stents in healthy arteries, but higher in diseased arteries. At 12 atm, the CoCr stent expanded the healthy and diseased arteries to an outer diameter of 3.85 ± 0.04 mm and 4.16 ± 0.1 mm, respectively, representing a 7% and 15.78% increase from the original outer diameter.

To further assess the uniformity of stent expansion, the standard deviation for the proximal, middle, and distal inner and outer diameters was calculated for both healthy and stenosed arteries. Both stents exhibited higher expansion uniformity in healthy arteries compared to stenosed arteries. The standard deviations were 0.09 mm and 0.089 mm in healthy arteries, and 0.11 mm and 0.13 mm in stenosed arteries for 316 L SS and CoCr stents, respectively. This indicates that while both stents maintain relative dimensional consistency in healthy arteries, stenosis increases variability along the stent length, highlighting the impact of external constraints on stent performance.

Upon deployment in healthy artificial arteries, the elastic recoil for 316 L SS and CoCr stents was 2.90% and 2.76% respectively, whereas higher recoil was observed in stenosed arteries (10.93% and 6.41%, respectively). During expansion in artificial arteries at maximum balloon pressure, the stent lengths decreased from initially 10.88 ± 0.05 mm to 10.65 ± 0.13 mm for 316 L SS stents, and from 10.82 ± 0.03 mm to 10.57 ± 0.23 mm for CoCr. Foreshortening in healthy artificial arteries was calculated as 0.94% for 316 L SS and 0.32% for CoCr stents, with slightly reduced foreshortening in stenosed arteries (0.78% and 0.17%, respectively). These results indicate that both stent types maintained dimensional stability, with 316 L SS stents exhibiting slightly higher recoil and higher foreshortening compared to CoCr stents.

### Implantability and expansion behavior of µ-LPBF stents in the rat model

Both 316 L SS and CoCr stents were successfully implanted *post-mortem* in the rat abdominal aorta. One 316 L SS stent was successfully implanted in a living rat. µ-LPBF stents could be processed through the femoral artery into the rat abdominal aorta and they were expanded with a commercial balloon catheter. As 316 L SS had a greater increase in diameter compared to CoCr stents with similar inflation pressure in the free expansion tests and in the silicone artery model, 316 L SS stents were implanted with 8 atm, whereas CoCr stents were implanted at 10 atm and 11 atm in the *post-mortem* setting.

**Fig. 6:**
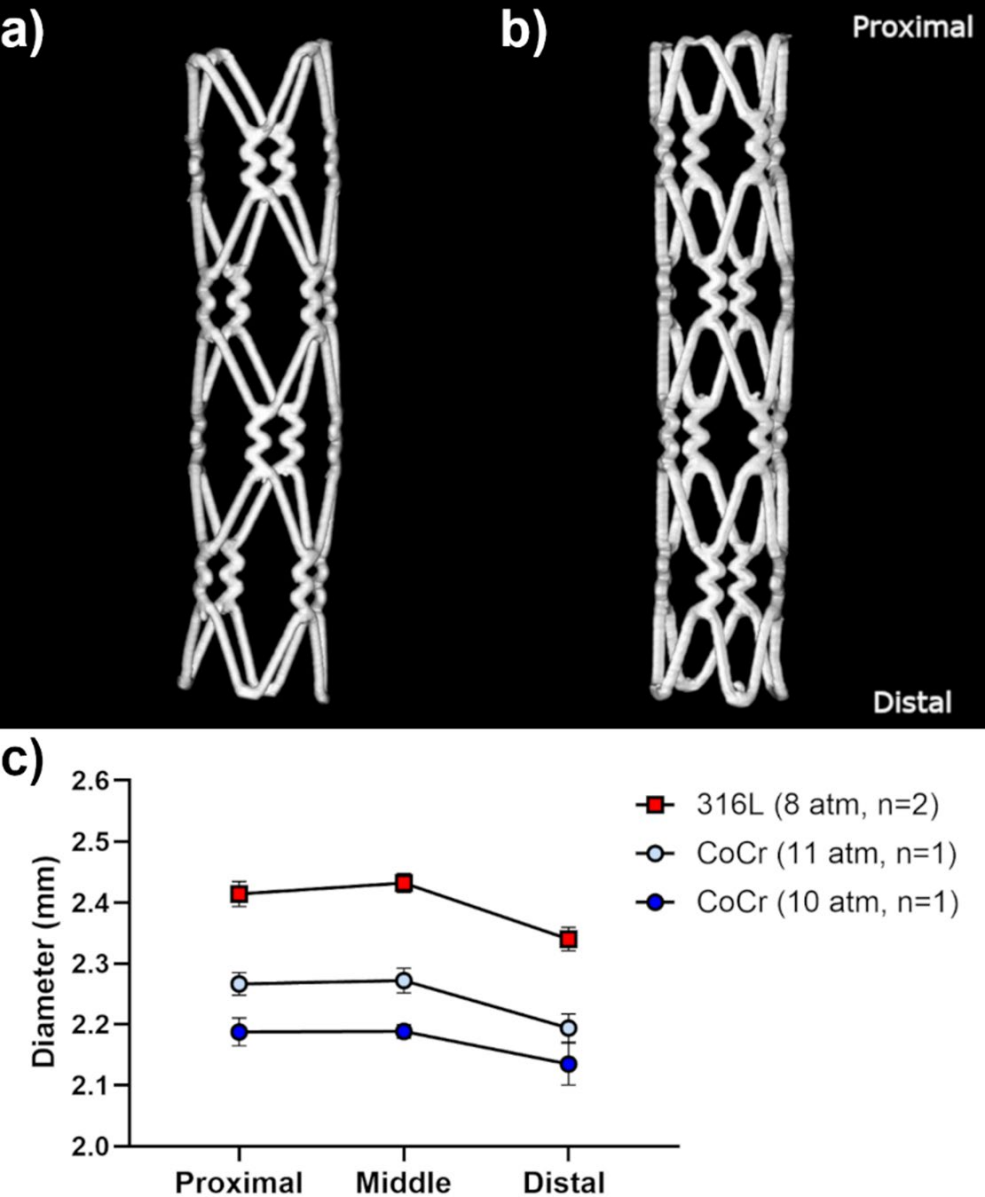
Deployed µ-LPBF stents in the rat aorta. (a) 3D geometry of a 316 L SS stent deployed at 8 atm, obtained from µCT scans. (b) 3D geometry of a CoCr stent deployed at 10 atm, obtained from µCT scans. (c) Mean diameter (± SEM) of deployed 316 L SS and CoCr stents, in the proximal, middle, and distal parts, based on µCT measurements.

µCT imaging of deployed µ-LPBF stents revealed no collapsing or cracking after stent deployment (Fig. 6a and b). 316 L SS stents deployed at nominal pressure exhibited greater expansion compared to the CoCr stent deployed at a higher pressure (Fig. 6a and b).

#### Post-mortem implantation

The expansion behavior of the µ-LPBF stents implanted *post-mortem* into the rat abdominal aorta was evaluated using µCT and histology. Mean diameters of 316 L SS and CoCr stents are shown in Table 1. 316 L SS stents implanted at nominal pressure (8 atm) exhibited mean diameters smaller than the desired diameter of 2.5 mm, as measured in both µCT and histology. Similarly, CoCr stents did not reach the desired diameter of 2.5 mm, even at higher pressures of 10 and 11 atm. This indicates an under-expansion of the µ-LPBF stents in the rat aorta. Notably, mean diameters measured by histology were smaller than those measured by µCT, possibly due to metal-induced radiation artifacts in µCT.

**Table 1:** Diameters of two 316 L SS and two CoCr µ-LPBF stents implanted *post-mortem* in the rat abdominal aorta using a 2.5 mm TREK balloon catheter, as assessed by µCT and histology.

Stent	Inflation pressure [atm]	Diameter of the 2.5 mm TREK Balloon [mm]**	Mean diameter ± SEM of stents in free-expansion [mm]	Mean diameter ± SEM (µCT) [mm]	Mean diameter ± SEM (histology) [mm]
316 L SS	8 *	2.51	2.39 ± 0.01	2.41 ± 0.02	2.15 ± 0.02
316 L SS	8 *			2.38 ± 0.02	2.17 ± 0.04
CoCr	10	2.58	2.37 ± 0.01	2.17 ± 0.02	1.92 ± 0.03
CoCr	11	2.60	2.39 ± 0.02	2.24 ± 0.01	2.04 ± 0.01

* Nominal pressure.

** At the given inflation pressure, according to manufacturer’s compliance chart.

Additionally, the mean diameter at the proximal, middle, and distal parts of the stents was measured from µCT images. The results show a non-uniform expansion of 316 L SS and CoCr stents, with the middle part showing the greatest expansion, followed by the proximal and the distal parts (Fig. 6c).

#### In vivo implantation

**Fig. 7:**
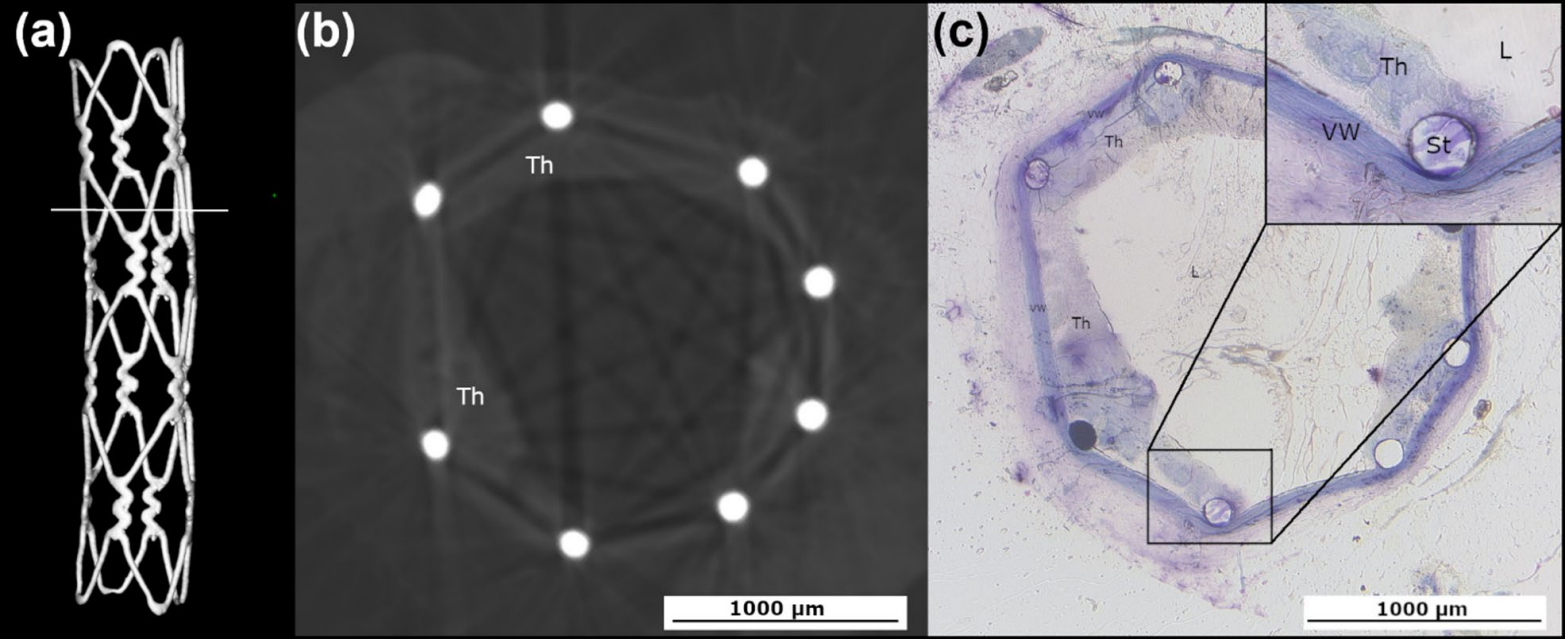
A 316 L SS stent implanted in a living rat. (a) 3D geometry of the deployed 316 L SS stent with a mark (white line) indicating the position of the cross-section in b and c. (b) Cross-sectional view of stented rat aorta from µCT. (c) Cross-sectional view of stented rat aorta from a histological image. *Th = thrombus*,* vw = vessel wall*,* and L = lumen.*

The 316 L SS stent was successfully implanted into a living rat. A 3D reconstruction of the deployed stent in µCT at 24 h post implantation is shown in Fig. 7a. Cross-sectional views of the stented aorta from µCT and histological images revealed a good stent strut apposition to the vessel wall. No apparent inflammatory response was observed around the stent struts. However, localized deformation of the vessel wall adjacent to stent struts indicated mild mechanical injury, with no severe vessel wall disruption identified. Thrombus formation was observed in the lumen (Fig. 7b and c), but importantly, no complete vessel occlusion occurred. The average lumen diameter measured from histological sections was 1.96 mm, which closely matched the 2.10 mm obtained from µCT.

### Expansion behavior of µ-LPBF stents in the rat model vs. in vitro free expansion

**Fig. 8:**
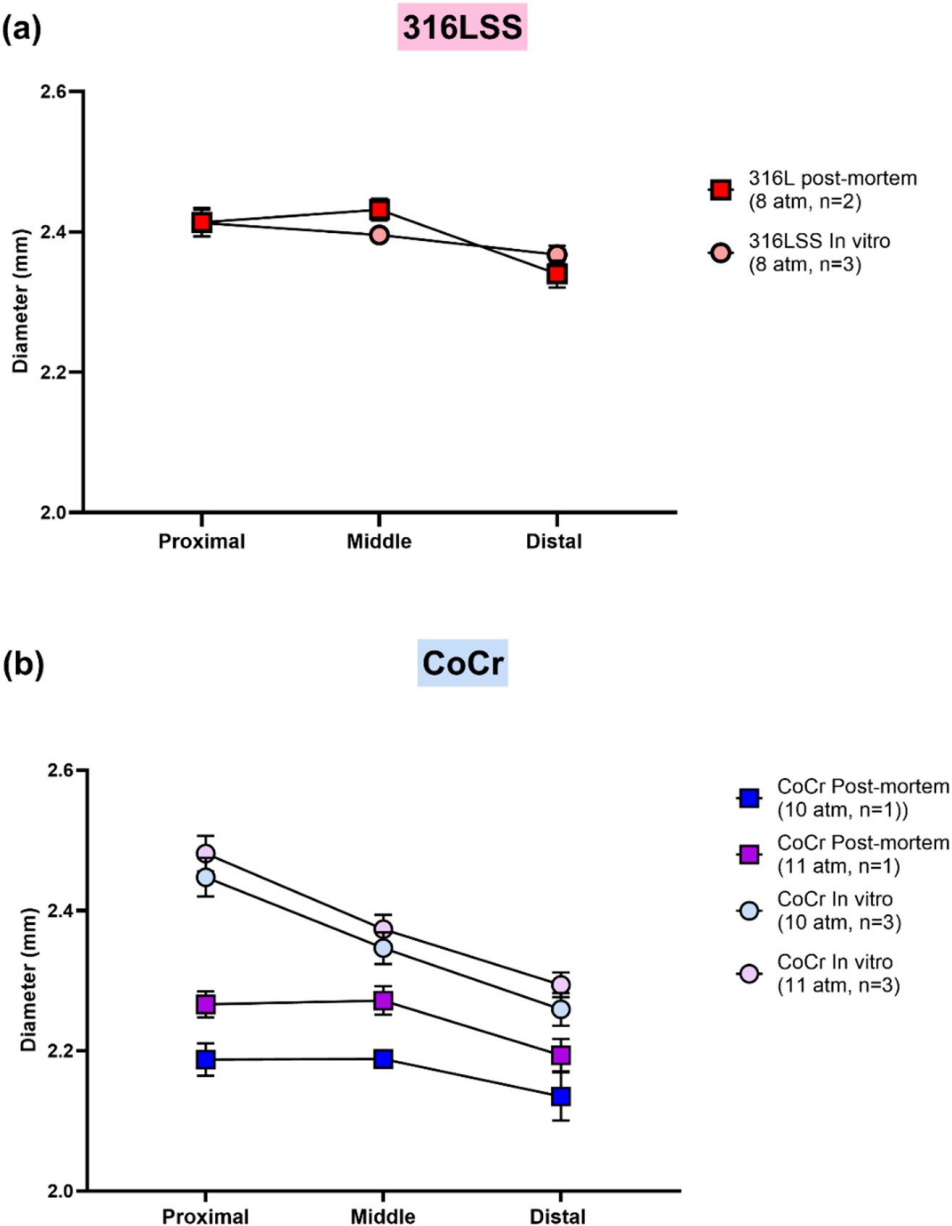
Mean diameter (± SEM) of the proximal, middle, and distal parts of µ-LPBF stents. (a) 316 L SS stents deployed *post-mortem* at 8 atm, compared with free-expanded stents. (b) CoCr stents deployed *post-mortem* at 10 and 11 atm, compared with free-expanded stents. Diameters of the *post-mortem* deployed stents were measured from µCT scans.

Mean diameters of the *post-mortem* deployed stent in rats were compared to freely expanded diameters at similar implantation pressure (Table 1). Additionally, the mean diameters at the proximal, middle, and distal parts of the free-expanded stents and deployed stents are presented in Fig. 8.

For 316 L SS stents, the mean diameters of freely expanded stents was close to those of implanted stents in rats at similar pressure (8 atm). In contrast, freely expanded CoCr stents revealed a larger diameter than CoCr stents implanted in rats at similar pressures (10 and 11 atm). Non-uniform expansion was observed in both experiments albeit with different patterns. Both µ-LPBF stents exhibited the largest diameter in the proximal part, followed by the middle and distal parts when freely expanded. In implanted µ-LPBF stents of each type, however, the greatest expansion was observed in the middle part.

## Discussion

### Feasibility of µ-LPBF stent implantation in animal models

Commercially available human coronary stents have been successfully implanted in rats for studies of in-stent restenosis^21,24,25^. However, to the best of our knowledge, no µ-LPBF stent has been implanted *post-mortem* or in vivo in an animal model. Most studies on µ-LPBF stents have focused primarily on stent design and in vitro mechanical performance. The implantability study is crucial, as a successful stent must not only have an optimal stent design and adequate mechanical properties but also be feasible to implant in the vessel with excellent flexibility and conformity. Therefore, the use of animal models is essential. The rat abdominal aorta is a well-established small-animal model for investigating vascular injury, stent implantation, and in-stent restenosis^21,25–27^. Its diameter of approximately 2 mm^28^ allows for the implantation of human-sized coronary stents.

In this study, µ-LPBF stents were successfully implanted in deceased rats and in one living rat. The balloon-mounted stents were delivered via the femoral artery over a guidewire, demonstrating adequate deliverability, as they could be advanced through the femoral artery to the abdominal aorta without deformation or fracturing. As confirmed by histology and µCT imaging, deployed µ-LPBF stents maintained structural integrity without collapsing under external tissue compression, indicating sufficient radial strength.

However, in the living rat, thrombus formation was observed along the stent struts one day post-implantation, which may be due to surface irregularities of the µ-LPBF stent^13,29^. As described in section “µ-LPBF stent manufacturing”, the as-printed µ-LPBF stents exhibit surface roughness in the range of 5–6 μm Ra, which is superior to previous as-printed SLM manufactured stents, with Ra values of 9.19 μm reported by Demir and Previtali^13^ and 8.4 μm reported by Finazzi et al.^30^. After electrochemical polishing, surface roughness improved to approximately 2 μm, which is close to the 1.09 μm reported for the commercial Multi-Link Vision CoCr stent^19^. However, this roughness remains higher than the recommended values for biomedical devices (< 0.5 μm)^29,30^. Nonetheless, this highlights a significant potential of µ-LPBF technology to surpass earlier generation of AM technologies. A recent study on µ-LPBF stents reported further improvement to 1.55 μm Ra after ECP^19^, suggesting that further optimization of post-processing may reduce thrombotic potential.

### Expansion behavior

In addition to the assessment of implantability and surface quality, the expansion performance was evaluated. Although both µ-LPBF stents were under-expanded at nominal balloon pressures, the 316 L SS stents consistently exhibited superior expansion compared to CoCr stents in free-expansion experiments, as well as in artificial arteries, and in the rat model. In free expansion, both types of µ-LPBF stents gradually increased in diameter with rising pressure, reflecting elastic-to-plastic deformation behavior.

For expansion in healthy artificial arteries, the 316 L SS stent diameter increased linearly between 2 and 3 atm (elastic region) with a reduced slope of the diameter-pressure curve after 3 atm (plastic yielding). CoCr stents showed similar elastic and plastic regions from 2 to 5 atm and 5–12 atm, respectively. In stenosed artificial arteries with 70% stenosis, the 316 L SS stent showed initial abrupt expansion, followed by gradual expansion, likely due to higher external constraints, while CoCr stents exhibited a more continuous expansion profile. With abrupt expansion, the stent diameter may be difficult to control, and the stent may potentially damage the vessel wall.

In addition, stent uniformity was evaluated. We observed non-uniform expansion of µ-LPBF stents in all experiments, with stents expanding unevenly along their length. Interestingly, unlike the typical ‘dog bone’ phenomenon – where both ends of a balloon-expanded stent expand more than the middle part^31–33^ – the middle part of µ-LPBF stents often expanded more than the ends. This pattern was evident in 316 L SS stents deployed in artificial arteries and consistent with CoCr stents under free expansion.

Moreover, the deviation in measured expansion between 316 L SS and CoCr stents was more pronounced in diseased than in healthy artificial arteries. The observed non-uniform expansion of µ-LPBF stents is likely due to small variations in strut thickness, surface roughness, and microstructure, including intragranular striations and deformation-induced phases from thermal stresses during printing, which locally reduce ductility but increase strength and radial resistance^19^.

The distal part consistently exhibited the smallest expansion, likely due to residual deformation induced by removal of the support legs from the construction platform during printing. Non-uniform stent expansion can create localized areas of higher stress at sites of greatest expansion leading to vessel wall injury, or malapposition at sites of lower expansion, potentially contributing to in-stent restenosis or thrombus formation^31–33^.

Balloon compliance strongly influences apparent stent expansion behavior. Non-compliant balloons may expand rapidly even at low pressures, however, according to the manufacturer’s compliance chart, the TREK balloon used in our study shows near-linear expansion from 2 to 12 atm. The apparent abrupt expansion observed is likely due to rapid unfolding of the balloon folds with stenosed or constrained vessels. Future studies should include balloon-pressure-diameter calibration to separate balloon effects from stent mechanical properties. However, the extent and morphology of lumen gain following stent deployment are largely determined by the stent design itself, as the stent relies on its plastic deformation to resist elastic recoil and maintain vessel patency.

### Comparison with commercial stents

Commercial stents such as Palmaz-Schatz (316 L SS) and Multi-Link Vision (CoCr) show highly uniform performance, with typical elastic recoil of 5–8% and foreshortening of approximately 1–2%^34,35^. In comparison, µ-LPBF stents exhibited recoil of 2.9% (316 L SS) and 2.76% (CoCr) in healthy artificial arteries, and slightly higher in stenosed models. Foreshortening in healthy artificial arteries was calculated to be 0.94% for 316 L SS and 0.32% for CoCr stents, with slightly reduced foreshortening observed in stenosed arteries (0.78% and 0.17%, respectively). While slightly underexpanded and showing non-uniform expansion along the stent length, µ-LPBF stents maintained acceptable lumen patency and structural integrity. These findings highlight the potential of µ-LPBF stents to achieve clinically relevant mechanical performance with further optimization of printing and post-processing parameters.

### Limitiations

This study represents a pilot investigation aimed at demonstrating the feasibility of µ-LPBF stent fabrication and implantation. Direct tensile and radial strength measurements were not performed. Instead, indirect indicators – including free expansion profiles, dimensional uniformity, and recoil rates – were used to infer material performance. Furthermore, due to limited sample availability, we did not perform any statistical comparisons between the two µ-LPBF stent types presented. Future studies are warranted to provide a full mechanical characterization of µ-LPBF stents.

Moreover, the commercial balloon catheter used in this study is not tailored to the µ-LPBF stents, which may have contributed to the observed underexpansion. However, we aimed to assess stent performance under standardized balloon conditions. Future studies need to optimize balloon-stent matching with balloon-pressure-diameter calibration to separate balloon effects from stent mechanical properties.

We used silicone artificial arteries to test stent expansion behaviour in tubular structures. Silicone arteries are generally slightly stiffer and less compliant than native vascular tissue, although we did not directly measure compliance in our experiment. This difference should be considered when interpreting the expansion and recoil behaviour observed in vitro.

There are also some inherent limitations of the rat abdominal aorta model when translating findings to human coronary applications. The arterial wall structure of rats is thinner than human coronary arteries, which may amplify local mechanical injury at high expansion pressures. In addition, rats lack atherosclerotic plaque, making it difficult to fully mimic the biomechanical environment of diseased human arteries. Despite these limitations, the rat model is an excellent model for the initial assessment of deliverability, expansion behaviour, and biocompatibility of novel stent technologies before progressing to larger animal models.

## Conclusions

We demonstrated the feasibility of µ-LPBF stent implantation in rats in both *post-mortem* and in vivo experiments. Both types of µ-LPBF stents were successfully implanted in rats with sufficient deliverability. In addition, µ-LPBF stents exhibited improved surface roughness after ECP compared to earlier generations of AM-manufactured stents.

Although non-uniform expansion behavior was observed, 316 L SS stents consistently outperformed CoCr stents in terms of expansion. While surface quality still requires further refinement to meet the recommended values for biomedical devices, and improvement in uniformity and expansion performance are needed, these results represent a meaningful step forward, particularly regarding implantability in an animal model. Although current µ-LPBF technology remains costly and is not yet fully mature for stent manufacturing, it represents a cutting-edge approach for the research and development of future-generation stents, paving the way for personalized strategies in percutaneous coronary intervention.

## Data Availability

The datasets generated and analyzed during the current study are part of ongoing investigations and are therefore available upon reasonable request to researchers who meet the criteria for access to confidential data. Requests for access should be directed to Priv.-Doz. Dr. med. Anne Turoni-Glitz, University Hospital Aachen, Department of Cardiology, Angiology, and Internal Intensive Medicine, aglitz@ukaachen.de.
